# Characterization of a novel corticosterone response gene in *Xenopus tropicalis* tadpole tails

**DOI:** 10.3389/fendo.2023.1121002

**Published:** 2023-01-26

**Authors:** Bidisha Paul, Rejenae Dockery, Valery M. Valverde, Daniel R. Buchholz

**Affiliations:** ^1^ Department of Biological Sciences, University of Cincinnati, Cincinnati, OH, United States; ^2^ School of Medicine and Health Sciences TecSalud Instituto Tecnológico y de Estudios Superiores de Monterrey (ITESM), Monterrey, Nuevo Leon, Mexico

**Keywords:** glucocorticoids, stress hormone, gene expression, *Xenopus tropicalis*, metamorphosis

## Abstract

Corticosteroids are critical for development and for mediating stress responses across diverse vertebrate taxa. Study of frog metamorphosis has made significant breakthroughs in our understanding of corticosteroid signaling during development in non-mammalian vertebrate species. However, lack of adequate corticosterone (CORT) response genes in tadpoles make identification and quantification of CORT responses challenging. Here, we characterized a CORT-response gene *frzb* (frizzled related protein) previously identified in *Xenopus tropicalis* tadpole tail skin by an RNA-seq study. We validated the RNA-seq results that CORT and not thyroid hormone induces *frzb* in the tails using quantitative PCR. Further, maximum *frzb* expression was achieved by 100-250 nM CORT within 12-24 hours. *frzb* is not significantly induced in the liver and brain in response to 100 nM CORT. We also found no change in *frzb* expression across natural metamorphosis when endogenous CORT levels peak. Surprisingly, *frzb* is only induced by CORT in *X. tropicalis* tails and not in *Xenopus laevis* tails. The exact downstream function of increased *frzb* expression in tails in response to CORT is not known, but the specificity of hormone response and its high mRNA expression levels in the tail render *frzb* a useful marker of exogenous CORT-response independent of thyroid hormone for exogenous hormone treatments and *in-vivo* endocrine disruption studies.

## 1 Introduction

Glucocorticoids (also known as stress hormones in vertebrates) dictate multiple events in vertebrate development including organ maturation and time of birth (1, 2). Lack of glucocorticoids during development can cause death at birth while excessive fetal exposure to glucocorticoids (from exogenous replacements or endogenous stress) can cause debilitating metabolic and neurological disorders later in adulthood (3–5). Glucocorticoids affect development directly and also indirectly by interacting with other hormones necessary for normal fetal development, such as thyroid hormone, insulin, and leptin (6–8). Although the indispensable role of glucocorticoids in vertebrate development has been known for decades, the molecular mechanisms underlying tissue-specific signaling pathways during direct and indirect actions of glucocorticoids on development are not well understood in vertebrates.

Amphibian metamorphosis is an exemplary model to investigate hormonal control of development in vertebrates due to conservation of thyroid and stress hormone signaling pathways between humans and frogs and due to their extreme dependence on both hormones for survival through metamorphosis (9–12). While much information about CORT (corticosterone, the main glucocorticoid in amphibians) signaling during frog metamorphosis has been obtained from gene knockout models, one of the main challenges of isolating and analyzing CORT responses in tadpoles is the lack of adequate and appropriate CORT response genes (13–15). The process of measuring CORT levels in the tissue or plasma is cumbersome and does not show whether altered CORT levels are having a physiological effect on tissues. Quantifying altered expression of a CORT-response gene provides an efficient and convenient way to assess CORT responsivity in tissues.

Previous studies have examined several CORT-response genes in tadpoles (16, 17). *klf9* is a well-established CORT-response gene, but it is also independently regulated by thyroid hormone (TH) (18, 19). Six CORT-response genes selected from a microarray study were used to establish a screening method for glucocorticoid endocrine disruption, but induction by thyroid hormone (TH) was not evaluated (20, 21). Corticotropin releasing hormone (CRH) is a common secretagogue for both CORT and TH in tadpoles, hence both CORT and TH levels increase during stress responses and during natural metamorphosis, such that a measure of increased *klf9* does not distinguish between a response due to CORT, TH, or both (22, 23). *ush1g* was identified in a microarray study as responsive to CORT, and its expression was subsequently characterized in tadpoles during development (17, 21). *ush1g* is induced by CORT and not TH and was thus considered to be a “CORT-only” gene, making it the only known specific marker of CORT action in tadpoles. However, although *ush1g* has a high fold-induction in response to exogenous CORT, change in *ush1g* expression is inconsistent among experiments during natural metamorphosis, which makes it an unreliable indicator of endogenous CORT responsiveness in tissues (13, 15).

In the current study, we have characterized frizzled related protein (*frzb*), which was identified as a CORT-only response gene in a global gene expression study where tadpole tails were treated *in vivo* and *in vitro* with vehicle, CORT, TH, and CORT+TH for 24 hours (24). Using *Xenopus tropicalis* and *X. laevis*, we validated the hormonal regulation of *frzb* by CORT and TH, quantified *frzb* expression in multiple tissues, identified the dose of CORT and duration of CORT treatment required for optimal *frzb* expression in tails, and measured it during natural metamorphosis. We also identified which corticosteroid receptor (glucocorticoid receptor, GR, and/or mineralocorticoid receptor, MR) is required for *frzb* expression.

## 2 Materials and methods

### 2.1 Animal husbandry

Lab reared, glucocorticoid receptor knockout (GRKO) *X. tropicalis* heterozygous mutants and wild-type *Xenopus tropicalis* adult male and female frogs were primed with 20IU of human chorionic gonadotropin (Sigma-Aldrich) in the evening and boosted with 200IU the following morning for breeding. Wild-type *Xenopus laevis* adult male and female frogs were injected with human chorionic gonadotropin (Sigma-Aldrich) at doses of 200IU and 600IU, respectively in the evening. Resulting tadpoles were reared at 26 degrees Celsius in reconstituted reverse osmosis water with water changes every 3 days, and tadpoles were fed powdered fry food (Sera Micron Nature) twice daily. Tadpoles from adult GR heterozygous crosses were genotyped into wild-type and GRKO homozygous mutants using a previously established heteroduplex mobility assay (13, 25). The use of animals in experiments was approved by the University of Cincinnati Institutional Animal Care and Use Committee (IACUC protocol # 21-06-21-01).

### 2.2 Selection of CORT-response genes

Buisine et al. (24) conducted global RNA-seq on tailfin skin of premetamorphic *Xenopus tropicalis* tadpoles treated with vehicle, 100 nM CORT, 10 nM triiodothyronine (T3), and CORT+T3. From their supplementary data, we selected genes that were significantly upregulated by CORT and CORT+T3 by 2-fold but were not induced by T3, resulting in identification of 7 genes that fit these criteria.

### 2.3 Hormone treatments and tissue harvest for gene expression

To identify gene(s) (out of 7 selected) with the highest fold change in response to CORT, we treated wild-type *X. tropicalis* premetamorphic tadpoles at Nieuwkoop and Faber (NF) 54 with 100 nM CORT and vehicle (ethanol) for 24 hours (26). To verify specificity of hormonal regulation by CORT and TH, wild-type *X. tropicalis* premetamorphic tadpoles (NF54) (n=10) were treated with vehicle (ethanol), 100 nM CORT, 10 nM T3, and 100 nM CORT plus 10 nM T3 by addition into the aquarium water. For the dose response experiment, NF54 WT *X. tropicalis* tadpoles were treated with vehicle, 50, 100, 250, and 500 nM CORT for 24 hours. For the time course, NF54 wild-type *X. tropicalis* tadpoles (n=10) were treated with 100 nM CORT for 0, 3, 6, 12, 24, and 48 hours. Water changes and hormone replacements were conducted daily. To investigate if GR is necessary for gene induction, we treated wild-type and GRKO NF54 *X. tropicalis* tadpoles with vehicle or 100 nM CORT. Control groups were treated with ethanol because most steroid hormones (including CORT) are dissolved in 100% ethanol. Hence, treating the control group with ethanol would exhibit possible effects of ethanol (if any) on gene expression in the tadpoles across all treatments. The treatment/hormone groups would then only demonstrate effect of CORT/TH treatment on gene expression. Tails (n = 10 per treatment) were harvested from MS-222-anesthetized tadpoles, snap frozen, and stored at -80 degrees Celsius until RNA isolation. To compare responsivity among tissues, brains (fore- plus midbrain portion including pituitary) and livers (n=10) from vehicle and CORT (100 nM) treated tadpoles were dissected (27) and stored as above. To determine gene expression during natural metamorphosis, tails (n=10) were harvested from tadpoles at premetamorphosis (NF54), pro-metamorphosis (NF58) and metamorphic climax (NF62) and stored as above.

### 2.4 Gene expression

RNA was extracted from frozen tissues using TRI REAGENT RT (Molecular Research Center, Inc., Cincinnati, OH) according to manufacturer’s instructions. Complementary DNA (cDNA) for each sample was synthesized from 1000 ng total RNA using the High- Capacity cDNA reverse transcription kit (Applied Biosystems). Quantitative PCR (qPCR) using 5uL of diluted cDNA was carried out using SYBR green master mix on a 7300 Real Time PCR System (Applied Biosystems) with gene-specific primers for *camta1, frzb, grpel1, klf9, musk, rpl8, sds, sult6b1, ush1g*, and *usp2* (**Table 1**). The reference gene ribosomal protein L8 (*rpl8*) was used and showed no significant differences among genotypes or treatments (data not shown). The relative quantification method ΔΔCt was used to compare expression levels of target genes normalized to the reference gene ribosomal protein L8 (rpl8) (28, 29).

**Table 1:** Primer and probe sequences of genes studied using quantitative PCR.

Gene	Species	Forward primer (5’-3’)	Reverse Primer (5’-3’)
*klf9*	*X. tropicalis*	TAAAGCCCATTACAGAGTCCAT	CACTCCTCATGAACCTCTTCTC
*klf9* *	*X. laevis* (L and S)	TACTGGGTGTGGCAAAGTTTAT	CTCTTCTCACAGAGTGGACATC
*camta1*	*X. tropicalis*	GGAGTGAAAGTCCTAATCACAGG	CAACAGAGTTGGAGAGAATCTGG
*frzb*	*X. tropicalis*	GAACAGATTCGATGCCAGACTT	CTTCATGGGCTTGCATTTACAG
*frzb*	*X. laevis* (L)	CTATCGTCACAGTGGAACAAGG	CCTTCATGGGCTTGCATTTAC
*frzb*	*X. laevis* (S)	GCTATCATCACAGTGGAACAAGG	CCTTCATGGGCTTGCATTTAC
*grpel1*	*X. tropicalis*	GAGTGAGACTCTTCAGGCAG	GGTTCTTACTCTTATCCTCGTCC
*musk*	*X. tropicalis*	CATCAGAGATCACATGGACAAGG	TCTACACTCAGAATGGTCAGGAG
*sds*	*X. tropicalis*	GTATTACCCAGATTCCGAAGGC	GAGCATACGGGCACTGTAA
*sult6b1*	*X. tropicalis*	ATGAAGAACCGTCTCCAAGAGT	ATGTATCCCAGGAGCTGTAGTTG
*usp2*	*X. tropicalis*	CCCACACTCTAAGAATACATGGC	GTATTACCCAGATTCCGAAGGC
*ush1g*	*X. tropicalis*	CTCTATGGGCGGCGTATC	GGAAGGAAAGGCAGTTCAGATG
*ush1g*	*X. laevis* (L)	CTTATCATGGGCACCTGGG	GGCTCCAAAGGACACCAAA
*rpl8*	*X. tropicalis*	CCACAATCCTGAAACAAAGAAA	CCTTGTATTTATGGTATGCACG
*rpl8*	*X. laevis* (L and S)	AGAAGGTCATCTCATCTGCTAAC	GGATAGGTTTGTCAATACGACCA

*F has 1 mismatch in S chromosome

### 2.5 Statistical analysis

Data were checked for normal distribution using Shapiro Wilk test of normality. For normally distributed data, unpaired Student’s t-tests, and full-factorial ANOVA were performed with base R (30). For data which did not follow normal distribution, non-parametric Kruskal-Wallis tests were conducted in R followed by pairwise comparisons using Wilcoxon rank sum exact tests. A p-value less than 0.05 was considered statistically significant.

## 3 Results

### 3.1 Selection of CORT-only genes and hormone specificity

**Figure 1:**
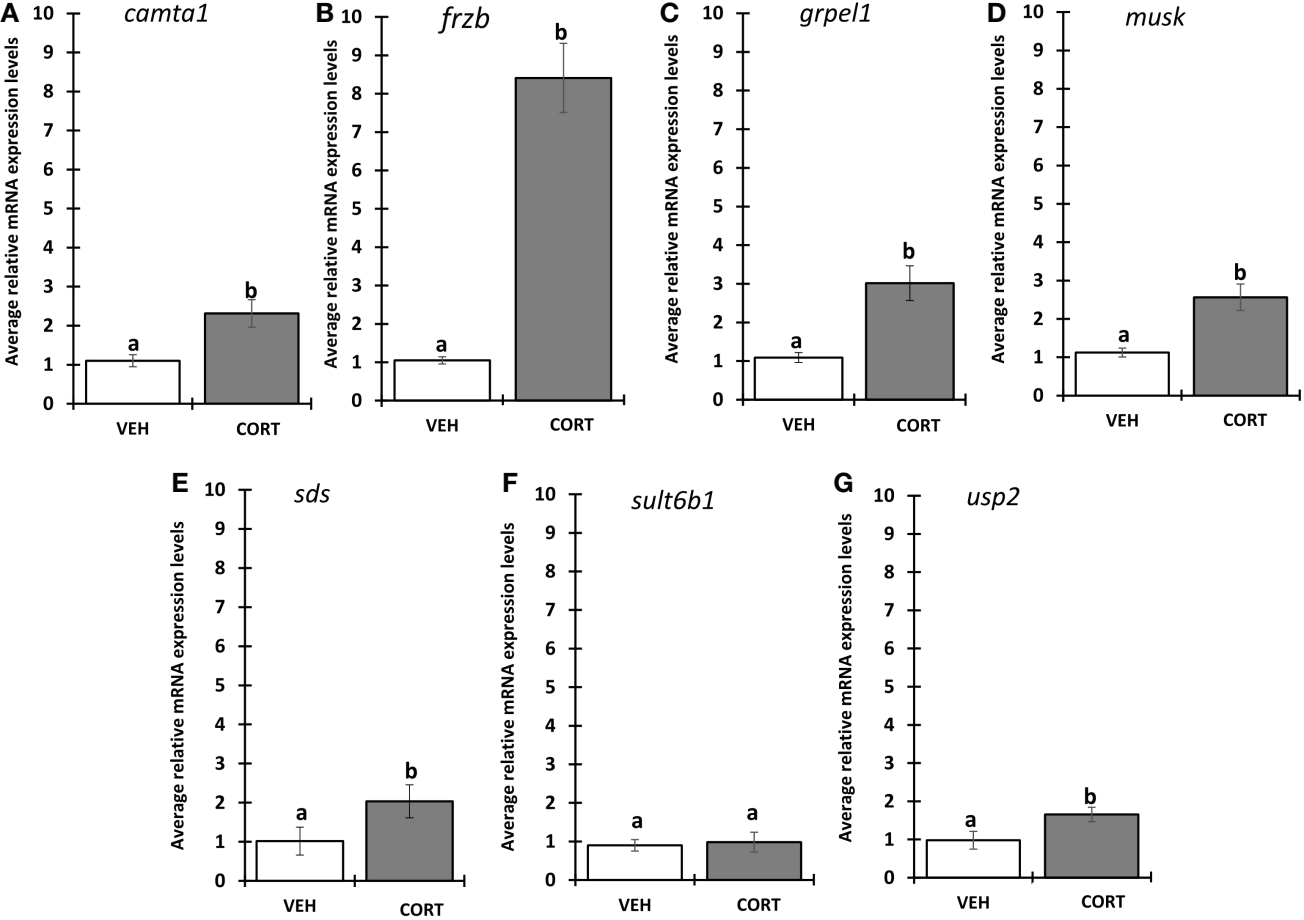
Evaluation of CORT response in selected genes. Premetamorphic tadpoles at Nieuwkoop and Faber stage 54 (NF54) were treated with vehicle control or 100 nM CORT (corticosterone) for 24 hours followed by tail RNA extraction and quantitative PCR to quantify mRNA expression using gene specific primers for **(A)** calmodulin binding transcription activator 1 (*camta1*), **(B)** frizzled related protein (*frzb*), **(C)** GrpE like 1, mitochondrial (*grpel1*) **(D)** muscle associated receptor tyrosine kinase (*musk*), **(E)** serine dehydratase (*sds*), **(F)** sulfotransferase family 6B member 1 (*sult6b1*), and **(G)** ubiquitin specific peptidase 2 (*usp2*). Bars represent mean mRNA levels relative to the housekeeping gene *rpl8* and normalized to a vehicle control sample. n = 10 tail samples per treatment. Error bars represent SE. Letters indicate significant groups, *p* < 0.05.

**Figure 2:**
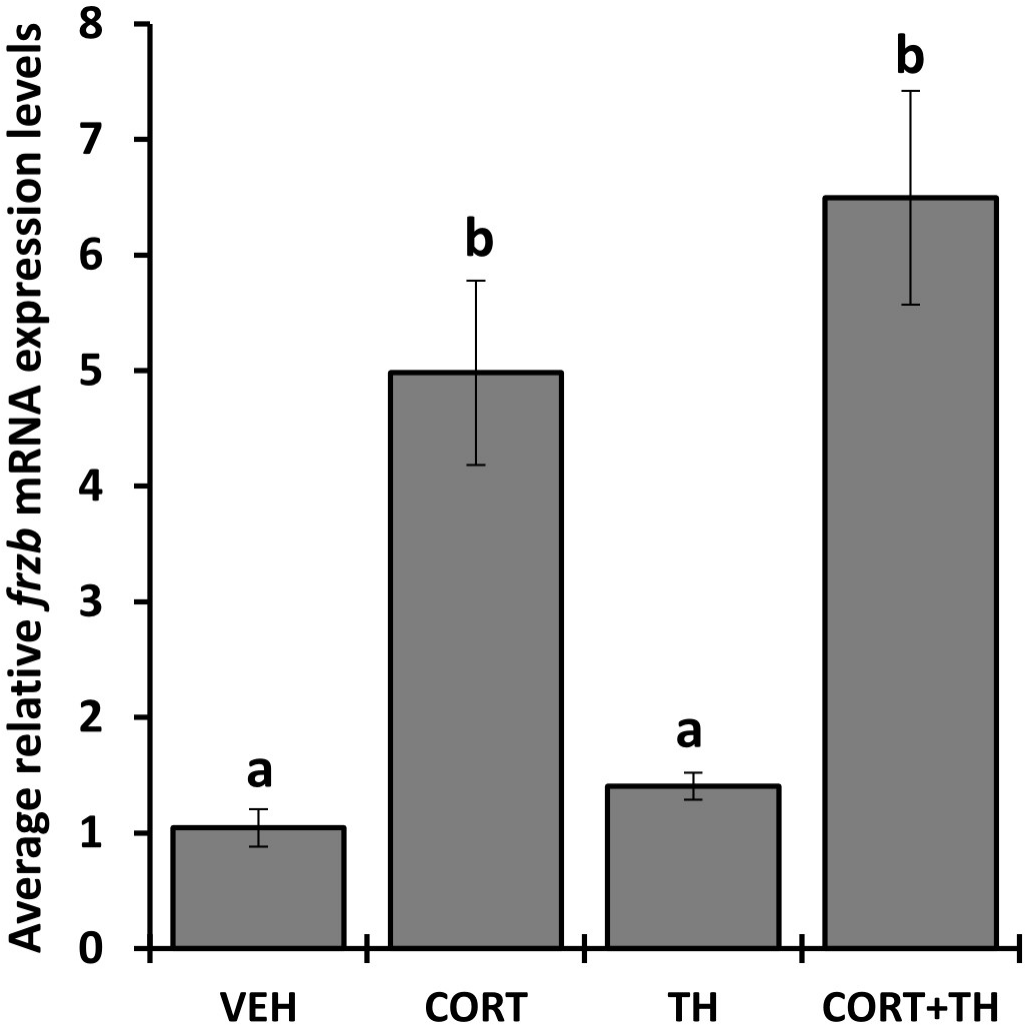
Hormone induction specificity of *frzb* in tadpole tails. Premetamorphic tadpoles (NF 54) were treated with vehicle, 100 nM CORT (corticosterone), 10 nM T3 (triiodothyronine), and CORT+T3 for 24 h. Tails were dissected, followed by RNA extraction and quantitative PCR for *frzb* and the housekeeping gene *rpl8*. Bars represent mean mRNA levels relative to *rpl8* and normalized to a vehicle control sample. n = 10 tail samples per treatment. Error bars represent SE. Letters indicate significant groups, *p* < 0.05.

We selected 7 genes induced by CORT and not by thyroid hormone (TH), i.e., “CORT-only” genes, from a previous RNA-seq study on premetamorphic tadpole tailfin skin (see Methods) (24), namely calmodulin binding transcription activator 1 (*camta1*), frizzled related protein (*frzb*), GrpE like 1, mitochondrial (*grpel1*), muscle associated receptor tyrosine kinase (*musk*), serine dehydratase (*sds*), sulfotransferase family 6B member 1 (*sult6b1*), and ubiquitin specific peptidase 2 (*usp2*), and retested their induction by CORT using quantitative PCR. Six of the seven genes, *camta1, frzb, grpel1, musk, sds*, and *usp2*, exhibited significantly induced mRNA levels in response to 100 nM CORT in the tails (**Figure 1**). We chose *frzb* for further validation and characterization due to high fold change (~8 fold) as compared to *camta1* (~2.3 fold), *grpel1* (~3 fold), *musk* (~2.5 fold), *sds* (~2 fold) and *usp2* (~1.6 fold) (**Figure 1**). To validate *frzb* as a CORT-only gene, we measured mRNA expression levels in response to thyroid hormone (TH). *frzb* was significantly induced by CORT and by CORT+TH but not by TH alone (**Figure 2**).

### 3.2 Tissue distribution, developmental profile, and receptor specific expression of *frzb*

**Figure 3:**
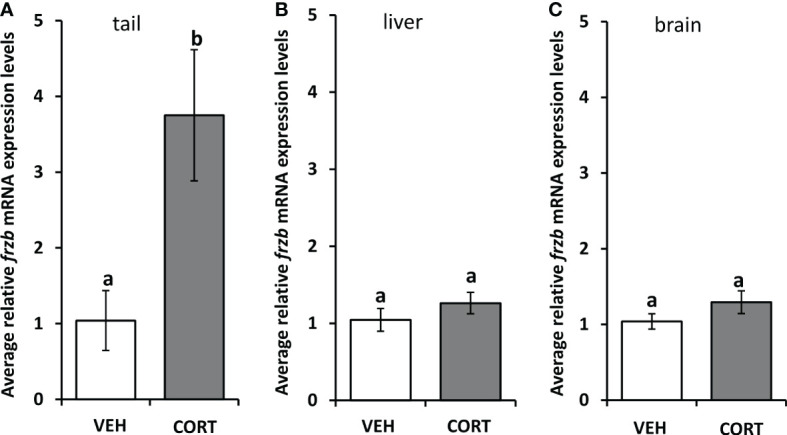
Tissue expression profile of *frzb* in tadpoles. Premetamorphic tadpoles (NF 54) were treated with vehicle and 100 nM CORT (corticosterone) for 24 hours. Tails **(A)**, livers **(B)**, and brains **(C)** were dissected, followed by RNA extraction and quantitative PCR to measure mRNA expression of *frzb* and the housekeeping gene rpl8. Bars represent mean mRNA levels relative to *rpl8* and normalized to a vehicle control sample. n = 10 tail samples per treatment. Error bars represent SE. Letters indicate significant groups, *p* < 0.05.

**Figure 4:**
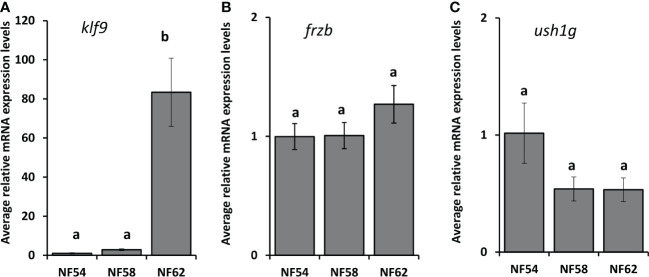
Developmental expression profile of *klf9, frzb*, and *ush1g* in tadpole tails throughout metamorphosis. Tails from tadpoles at the indicated NF stages were harvested, followed by tail RNA extraction and quantitative PCR to measure mRNA expression levels of *klf9* **(A)**, *frzb* **(B)**, *ush1g* **(C)** and the housekeeping gene *rpl8*. Bars represent mean mRNA levels relative to *rpl8* and normalized to a vehicle control sample. n = 10 tail samples per treatment. Error bars represent SE. Letters indicate significant groups, *p* < 0.05.

**Figure 5:**
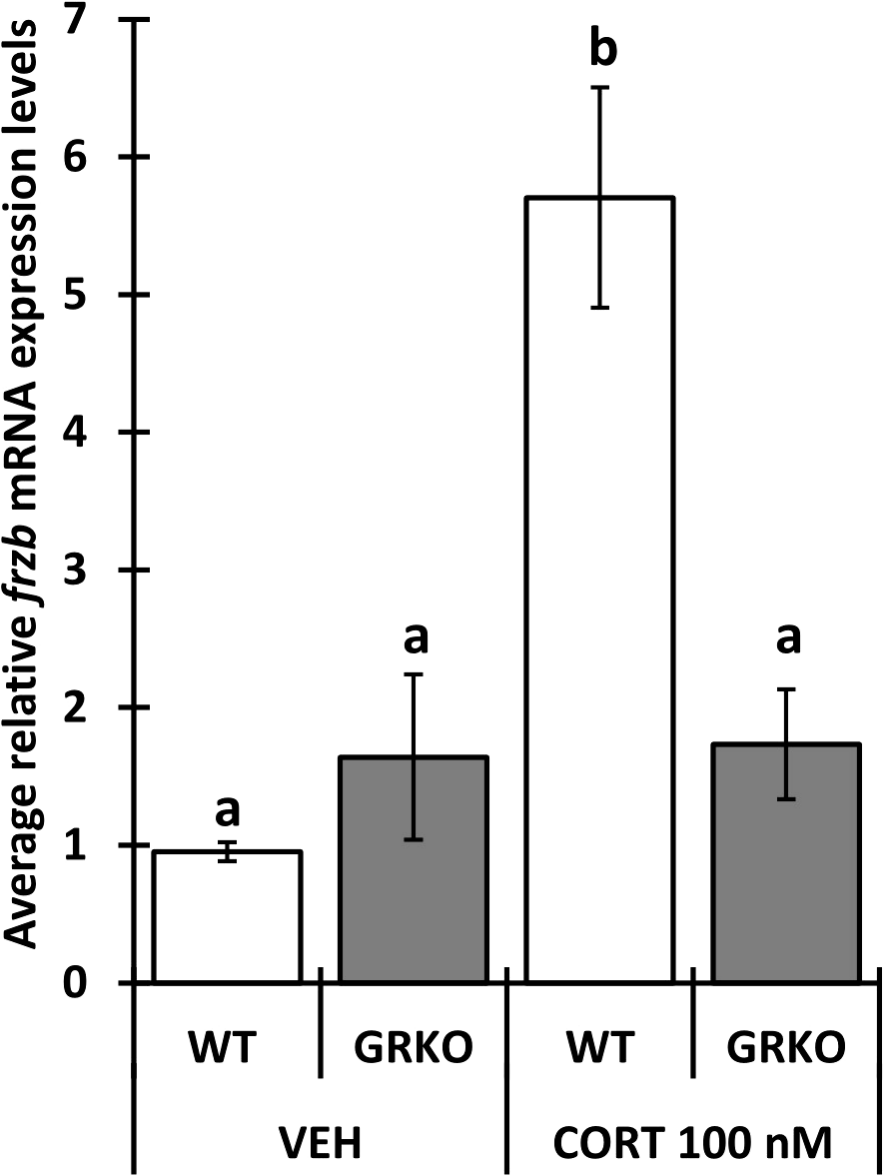
Impaired *frzb* induction in glucocorticoid receptor knockout (GRKO) tails. Premetamorphic wild-type and GRKO tadpoles (NF 54) were treated with vehicle control or 100 nM CORT (corticosterone) for 24 hours followed by tail RNA extraction and quantitative PCR to quantify mRNA expression of *frzb* and the housekeeping gene *rpl8*. Bars represent mean mRNA levels relative to *rpl8* and normalized to a vehicle control sample. n = 10 tail samples per treatment. Error bars represent SE. Letters indicate significant groups, *p* < 0.05.

To assess the ability to use *frzb* to quantify tissue-specific CORT responses in tadpoles, we measured *frzb* induction after CORT treatment among tissues. When NF54 tadpoles were treated with 100 nM CORT for 24 hours, only tails and not livers and brains demonstrated a significant increase in *frzb* expression levels (**Figures 3A–C**). We then measured *frzb* expression levels just in tails during natural metamorphosis where endogenous CORT levels peak at the climax of metamorphosis. Even though *klf9* was highly induced at NF 62 (**Figure 4A**), we found that *frzb* expression did not change significantly during natural metamorphosis (**Figure 4B**). The only previously known CORT-only gene *ush1g* also was not induced during natural metamorphosis (**Figure 4C**). Receptor specificity of CORT-response genes is important for many types of studies, and so far, all known CORT-response genes are induced by GR only (i.e., not by mineralocorticoid receptor). Here, we determined if GR is required for *frzb* expression. Upon CORT treatment, tails from wild-type tadpoles exhibited significantly higher *frzb* expression, but tails from CORT-treated GR knockout tadpoles showed that *frzb* expression was not significantly different from vehicle-treated wild-type or GR knockout tails (**Figure 5**).

### 3.3 Dose response and time course of *frzb* induction in tails

**Figure 6:**
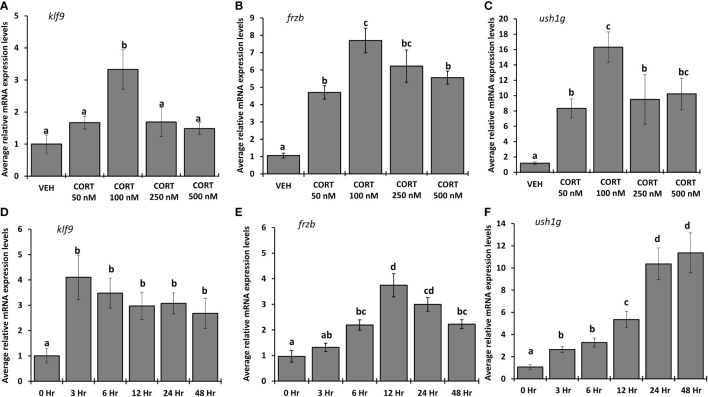
Dose response and time course for CORT induction of *frzb, ush1g*, and *klf9* in tadpole tails. **(A-C)** Premetamorphic tadpoles (NF 54) were treated with vehicle and 50, 100, 250, and 500 nM of CORT (corticosterone) for 24 hours. **(D-F)** Premetamorphic tadpoles (NF 54) were treated with 100 nM CORT for 0, 3, 6, 12, 24, and 48 hours. Tails were dissected from tadpoles followed by RNA extraction and quantitative PCR to measure mRNA expression of *frzb, ush1g*, and *klf9* and the housekeeping gene *rpl8*. Bars represent mean mRNA levels relative to *rpl8* and normalized to a vehicle control sample. n = 10 tail samples per treatment. Error bars represent SE. Letters indicate significant groups, *p* < 0.05.

To determine the dose of CORT required for maximum induction of *frzb*, we measured *frzb* mRNA expression in NF54 tadpole tails after treating with vehicle (ethanol), 50, 100, 250 and 500 nM CORT for 24 hours (**Figures 6A–C**). *frzb* was significantly induced at 50 nM CORT, but the highest fold change (~7 times) occurred in the 100 nM CORT treatment (**Figure 6B**). The induction of *frzb* by higher doses of CORT showed decreasing levels with increasing doses of treatment indicating an inverted “U-shaped” dose response curve (**Figure 6B**). The other two CORT-response genes *klf9* and *ush1g* showed similar dose response curves (**Figures 6A, C**). To determine the duration of CORT treatment which results in highest mRNA expression levels of *frzb*, we measured *frzb* expression in NF54 tadpole tails after treating with 100 nM CORT for 0, 3, 6, 12, 24, and 48 hours (**Figures 6D–F**). *frzb* was significantly induced at 6 hrs. after CORT treatment, but the highest expression level occurred at 12 hrs. and stayed significantly higher at 24 hrs., after which it was significantly lower at 48 hrs compared to peak expression (**Figure 6E**). We compared time course of *frzb* induction to *klf9* and *ush1g* and found that *klf9* showed the highest induction at 3 hours and stayed significantly high at 48 hours. *Ush1g* induction began at 3 hours and kept increasing throughout 24 hrs (**Figures 6D, F**).

### 3.4 *Frzb* induction in *X. laevis* tadpoles

**Figure 7:**
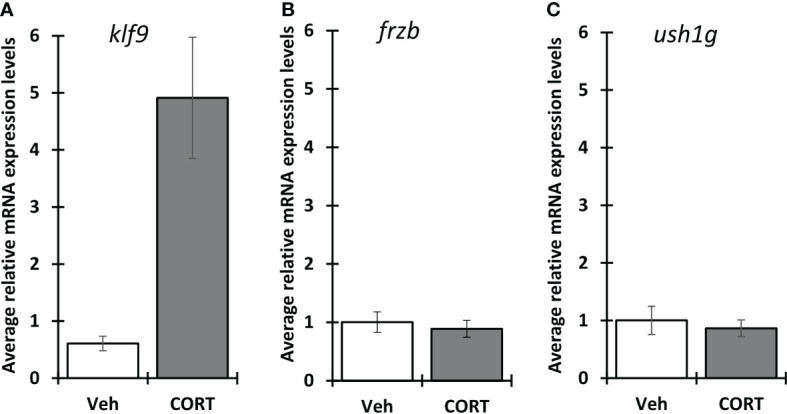
Induction of *klf9*, *frzb* and *ush1g* in *X. laevis*. Premetamorphic (NF 54) *X. laevis* tadpoles were treated with vehicle control or 100 nM CORT (corticosterone) for 24 hours followed by tail RNA extraction and quantitative PCR to quantify mRNA expression of *klf9* **(A)** *, frzb* **(B)**, and *ush1g* **(C)**. Bars represent mean mRNA levels relative to the housekeeping gene *rpl8* and normalized to a vehicle control sample. n = 10 tail samples per treatment. Error bars represent SE. Letters indicate significant groups, *p* < 0.05.

Because *X. tropicalis* and *X. laevis* are often interchangeably used for studying thyroid and stress hormone signaling, we wanted to know if *frzb* is induced in *X. laevis* as well (**Figures 7A–C**). Surprisingly, contrary to *X. tropicalis* tails, 100 nM CORT did not induce *frzb* nor *ush1g* in NF54 *X. laevis* tails (**Figures 7B, C**), even though we found that *klf9* was induced (**Figure 7A**). We tried primers sets for both chromosomes, i.e., two primer sets for *frzb.L* and one for *frzb.S*, all with similar results (data not shown).

## 4 Discussion

Corticosteroids have pleiotropic effects on vertebrate development directly and/or indirectly through interaction with other key hormones, mainly thyroid hormone, insulin, and leptin (31–34). While much has been known about corticosteroid signaling from mammalian models, questions remain about how corticosteroid deficiency and excess in early development increase the risk of metabolic and neurological diseases in adults (4, 35). Lower vertebrates such as fish and frogs have been instrumental in unfolding some of the key corticosteroid signaling mechanisms, especially through the use of knockout models (13–15, 36, 37). Investigations on CORT signaling in tadpoles have also informed us a great deal about the impact that adaptation to stressful stimuli can have on the evolutionary ecology of the species (38). However, lack of adequate CORT response genes makes it challenging to detect and quantify endogenous and exogenous CORT response without going through the expensive and labor-intensive process of quantifying steroid hormone in the tissue and/or plasma. In this study, we characterized *frzb* (frizzled related protein), which along with 6 other genes (*musk, camta1, sult6b1, sds*, and *grpel1)* were found to be induced by only CORT and not TH in a previous global RNA-seq study on the *X. tropicalis* tail skin. This unique pattern of hormone induction drove our interest in these CORT-only genes. Previously, *klf9* and *ush1g* had been the only 2 CORT-response genes reported in tadpoles (16–18). However, *klf9* is also induced independently by TH and synergistically by both CORT and TH (19). *ush1g* is a CORT-only gene, however, *ush1g* expression during natural metamorphosis is inconsistent between clutches and thus unreliable (13, 15).

We further verified *frzb* to be induced only by CORT independent of TH with a fold change of around 8, which was higher than the remaining 5 genes, which had a fold change up to 2.5. Due to higher fold change, we used *frzb* for further characterization of a CORT-only response gene. We found that *frzb* expression was highest in the tails and no change in *frzb* expression was observed in the brain and liver. *Frzb* expression patterns in the brain and liver are similar to our previous study reporting *ush1g* expression in the brain and liver. No change in brain *ush1g* expression was observed and there was small increase in liver *ush1g* expression in response to CORT treatment (17). *Klf9* levels were previously found to be induced in the brain by CORT treatment (16). Highest *frzb* expression in the tails took place at 12-24 hours when treated with 100-250 nM CORT. Similar to *frzb*, both *klf9* and *ush1g* expression levels peak at 100 nM in the dose response experiment in the current study. In the time course experiment, similar to *frzb*, *ush1g* peaks at 24 hours. However, *klf9* expression levels peak at 3 hours in the current study and at 2 hours in a previous study probably because *klf9* is a direct CORT response gene (16).

In embryos, *frzb* is expressed in the anterior endoderm or in the prechordal mesoderm and plays a crucial role in anteroposterior patterning in *Xenopus laevis* by binding and inactivating Xwnt-8 during gastrulation (39). Inhibition of Wnt-8 signaling is necessary to prevent excessive ventralization and posteriorization by Wnt/β-catenin signaling in order to promote dorsoanterior development, specifically head formation (40, 41). *frzb* is also known to promote cartilage and long bone development in chick embryos (42, 43). However, there is no evidence of *frzb* affecting development in tadpoles through alteration of corticosteroid signaling. We showed *frzb* induction by CORT requires GR, and lack of GR in GR knockout tadpoles, which exhibit altered developmental rate and death at metamorphosis, had no apparent effect on head formation. Future studies using *frzb* knockout tadpoles should be conducted to identify an effect of *frzb* in CORT-induced tadpole tails.

Two surprising results from characterizing *frzb* were no increase in expression levels during natural metamorphosis and no induction by exogenous CORT in *X. laevis* despite high level of induction in *X. tropicalis*. An explanation could be that the measurable increases in CORT levels during metamorphic climax might be enough to induce *klf9* (an early and direct response gene), but not enough to induce *ush1g* and *frzb* (44, 45). Additionally, TH levels also increase during metamorphic climax, hence, a combination of both CORT and TH might be enough to increase expression levels of the CORT and TH response gene *klf9* but not CORT-only genes *frzb* or *ush1g* (23)*. frzb* is expressed in both *X. tropicalis* and *X. laevis*, although due to allotetraploidy, *X. laevis* also has two copies (each on L and S chromosomes) of *frzb* (46). Primers quantifying expression levels of *frzb* in this study were designed to target L and S chromosomes which should have captured change in expression levels induced by one and/or the other allele of *frzb.* However, despite strong conservation in gene expression between *X. laevis* and *X. tropicalis*, significant differences have been observed in gene expression patterns between the two related species (47–49). Additionally, the role of *frzb* in anteroposterior patterning in *Xenopus* has mostly been studied in *X. laevis* tadpoles and not X. *tropicalis*, and no previous information exists regarding whether *frzb* is regulated by CORT in either of the two related species (39, 50–52). Further investigation is required surrounding the role of *frzb* in CORT signaling and causes of such species-specific divergence.

## 5 Conclusion

In this study, we have reported a glucocorticoid response gene, frizzled related protein (*frzb*) in *X. tropicalis* (but not *X. laevis*) tadpoles, which exhibit high mRNA induction in response to exogenous CORT but not TH in tails, and hence can be used to detect and quantify CORT responsivity independent of TH. Unfortunately, *frzb* cannot be used to study changes in CORT levels during natural metamorphosis or to study CORT response in the liver or brain. *frzb* is induced through GR and not MR and thus could be used to evaluate potential endocrine disrupting chemicals targeting GR. Any potential effects of *frzb* in CORT signaling await further study.

## Data availability statement

The raw data supporting the conclusions of this article will be made available by the authors, without undue reservation.

## Ethics statement

The animal study was reviewed and approved by University of Cincinnati Institutional Animal Care and Use.

## Author contributions

BP: Conceptualization, Funding acquisition, Investigation, Methodology, Writing – original draft, Writing – review and editing. RD: Sample collection, RNA extraction and quantitative PCR. VV: Sample collection, RNA extraction and quantitative PCR. DB: Conceptualization, Funding acquisition, Project administration, Supervision, Writing – review and editing. All authors contributed to the article and approved the submitted version.
